# UAV Small Target Detection Model Based on Dual Branches and Adaptive Feature Fusion

**DOI:** 10.3390/s25154542

**Published:** 2025-07-22

**Authors:** Guogang Wang, Mingxing Gao, Yunpeng Liu

**Affiliations:** 1 College of Information Engineering, Shenyang University of Chemical Technology, Shenyang 110142, China; mingxinggao99@163.com; 2 Shenyang Institute of Automation, Chinese Academy of Sciences, Shenyang 110016, China; ypliu@sia.cn

**Keywords:** drone, target detection, YOLOv8, adaptive feature fusion, large separable kernel attention

## Abstract

In order to solve the problem of small and dense targets in drone aerial images, a small target detection model based on dual branches and adaptive feature fusion is proposed. The model first constructs a small target detection framework with dual branches to improve the detection accuracy while reducing the number of parameters. Secondly, the model introduces semantic and detail injection (SDI) in the neck network and embeds bidirectional adaptive feature fusion in the detection head to innovate and optimize the feature fusion mechanism, achieve the full interaction of deep and shallow information, enhance the feature representation of small targets, and overcome the problem of scale inconsistency. Finally, in order to focus on the target area more accurately, we introduce the large separable kernel attention mechanism into the convolutional layer to provide it with a richer and more comprehensive feature representation, which significantly improves the detection accuracy of targets of different scales. The experimental results show that the model algorithm performs well in the VisDrone2019 dataset. Compared with the original model, the mAP50 of this model increases by 20.9%, the mAP50–95 increases by 23.7%, and the total number of parameters decreases by 61.3%, making it more suitable for drones.

## 1. Introduction

**Figure 1:**
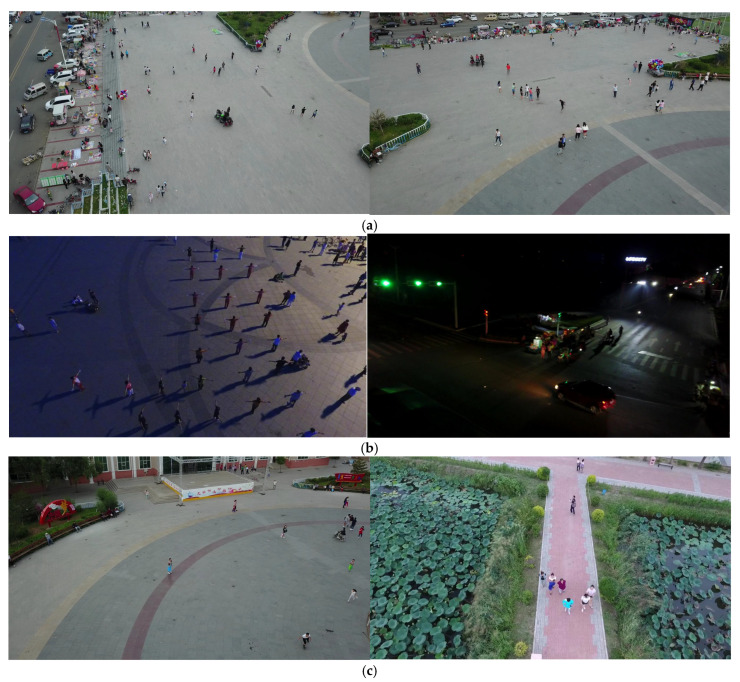
Drone aerial pictures: (**a**) intensive aerial photography scenes; (**b**) night aerial pictures; (**c**) small objects.

In recent years, drones have played an important role in many fields due to their excellent flexibility, maneuverability, and accurate perception of targets. Combining deep learning-based target detection methods with drone systems has become one of the current research and application focuses. Since drones are affected by the perspective and flight altitude during navigation, the targets in drone aerial images are generally small. At the same time, drones are easily affected by the environment during navigation, resulting in blurred aerial images, making target detection in aerial photography more difficult. Figure 1 is a drone aerial photo. Therefore, accurately detecting and identifying small targets in drone navigation is a challenging direction.

Before the development of deep learning, target detection mainly relied on traditional methods such as Histogram of Oriented Gradient (HOG) [1], Scale Invariant Feature Transform (SIFT) [2], and Local Binary Pattern (LBP) [3]. However, for targets with rich textures, variable shapes, or occlusion, the expression ability of manual features is limited, resulting in low detection accuracy. With the application of deep learning in target detection, target detection has been fully developed. The mainstream target detection in the field of deep learning is divided into two categories: single-stage detection algorithms and two-stage detection algorithms. Two-stage target detection algorithms mainly include R-CNN [4], Faster R-CNN [5], FPN [6], etc., which have good performance in target detection accuracy. However, since they need to generate pre-selected boxes of possible targets before detection and classification, they have a large number of parameters, slow processing speed, poor real-time performance, and are not suitable for application on drones. On the contrary, single-stage target detection algorithms mainly include SSD [7], RetinaNet [8], YOLO [9], and other series of algorithms, which have good performance in real-time target detection and are widely used on drones, especially the YOLO series. However, the article in [10] points out that such algorithms often need to sacrifice mAP in exchange for inference speed, while the two-stage algorithm sacrifices speed to achieve higher accuracy; in addition, the article in [11] found that the two-stage algorithm and the Transformer model can more accurately capture the target context relationship in complex scenes through a step-by-step optimization mechanism, and its average positioning accuracy is significantly higher than that of the YOLO series. This makes the detection accuracy of the YOLO series different from that of the two-stage algorithm, and also prompts the industry to explore how to improve detection accuracy while ensuring real-time performance.

From the existing research, Dan Munteanu et al. [12] compared the performance of YOLOv5, SSD, and EfficientDet in sea mine detection and found that YOLOv5 achieved 80.3% mAP50 at 15.8 GFLOPs computing power. Its generalization ability in small target detection and complex sea conditions was significantly better than other models, verifying the efficiency advantage of single-stage algorithms in real-time detection of drones. Coincidentally, in the field of biological control, Danilo Oliveira and Samuel Mafra [13] integrated YOLOv7 and LoRaWAN communication technology into the intelligent trap system, and achieved 97% detection accuracy of Aedes aegypti through dynamic ventilation capture and multi-scale feature fusion design, providing an engineering paradigm for target detection of IoT edge devices. In addition, the IoT mobile sensor unit developed by Dhou et al. [14] achieved 99% classification accuracy in obstacle recognition for visually impaired people through multi-source data fusion, such as accelerometers and ultrasonic sensors, and support vector machine classification. Its sensor collaboration strategy provides a reference paradigm for multi-modal data fusion of drones.

In terms of transmission and detection collaborative optimization, the LoRaWAN quasi-real-time video surveillance unit built by Fort et al. [15] controlled the image transmission delay within 10 min through WebP compression and block transmission strategy, alleviating the efficiency problem of low-power wide area networks in bandwidth-constrained scenarios. However, the study pointed out that when the image resolution is higher than 320 × 240, the feature attenuation caused by compression will reduce the detection accuracy by 18.3%. Pronello and Garzón Ruiz [16] revealed the performance differences of commercial automatic passenger counting (APC) systems in a field test; the stereoscopic vision solution with a claimed 98% accuracy only achieved 53.11% boarding detection accuracy during peak hours, while the low-cost Raspberry Pi solution based on YOLOv5 showed an advantage with an accuracy of 72.27%. This result reveals that the traditional model has the problem of insufficient feature representation and failure of multi-target association in dense target occlusion scenarios. Based on existing research, more scholars have explored different technical paths. Wei Zhan et al. [17] used the KMeans++ clustering algorithm to redesign the anchor box size to improve the matching probability between the target box and the anchor box in the drone shooting scene, and improved the detection accuracy of the model without increasing the computational overhead. Krzysztof Gromada et al. [18] proposed a new method combining YOLOv5 with classical image processing post-processing. The method used classical image processing methods to determine the minimum area rotation bounding box, and combined with the pixel resolution information in the SAR image metadata, the object size was compared with the acceptable range to reduce the misclassification situation. It is used for real-time target detection and recognition of drone-mounted SAR.

Among recent advances, the RSUD20K dataset [19], published in 2024, focuses on road scene object detection for autonomous driving. It provides a benchmark for small target detection in complex road scenarios and shows that even state-of-the-art detectors struggle with small, occluded targets, consistent with the challenges in UAV scenes. However, RSUD20K differs fundamentally from UAV aerial scenarios. It targets driving-view road scenes with relatively large targets and simple backgrounds, while our UAV scenes involve tiny targets, complex backgrounds, and perspective-induced occlusion. This scene difference means RSUD20K-adapted methods cannot directly apply to UAVs, highlighting the need for our UAV-specific optimization.

Although the above research has made some progress, the current UAV small target detection still faces three core challenges: (1) Insufficient small target feature extraction capabilities, such as the missed detection rate of such targets in reference [12], exceeding 40%. (2) The low efficiency of multi-scale feature fusion in dense scenes leads to a sharp drop in detection accuracy under complex working conditions in reference [16]. (3) Resource competition between low-power transmission and high-precision detection, such as the need for LoRaWAN to achieve a balance between accuracy and real-time performance at the 4.3M parameter level in reference [13].

To address these issues, a systematic solution from architecture reconstruction to module innovation is proposed. The main contributions are as follows: (1) The topological innovation of the dual-branch lightweight skeleton achieves the coordinated optimization of parameter quantity and feature retention by optimizing the network architecture. (2) The cross-mechanism fusion of the semantic detail injection module adopts a cross-mechanism fusion strategy to solve the information attenuation problem in the traditional feature fusion process. (3) A bidirectional adaptive spatial feature fusion mechanism is designed to dynamically allocate fusion weights according to the target scale, thus improving the detection robustness of multi-scale targets. (4) The C2f_LSKA unit is constructed to achieve the dual gains of “architecture burden reduction–attention focus” in scenarios with limited computing power, especially significantly improving the detection accuracy of small targets with dense occlusion.

The existing improved models based on YOLO and the detection scheme proposed in this paper have common optimization goals: both focus on improving the performance of small target detection in drones and adopt multi-scale feature processing strategies to try to balance detection accuracy and real-time performance.

However, there are essential differences in the technical paths. Existing models, such as in reference [17], use KMeans++ clustering to optimize the anchor box to improve the target matching probability, but retain the P2–P5 full-scale feature pyramid, and do not solve the redundancy problem of the P5 layer for small target representation; the dual-branch architecture of this paper actively removes the P5 layer. To compensate for the feature loss that may be caused by this operation, a dual-branch architecture of the backbone and neck and backbone and head is constructed, and feature extraction is focused on the P2–P4 high-resolution layer, achieving a significant reduction in parameter scale. The design differences of the feature fusion mechanism are even more significant. Traditional methods rely on unidirectional feature pyramids or post-processing, which have the contradiction between cross-layer information attenuation and computational overhead. The BASFF module proposed in this paper integrates bidirectional paths and adaptive weight allocation, solves the scale inconsistency problem of dense scenes through dynamic weighting, and reduces the computational complexity by 38% compared with traditional ASFF. At the level of attention mechanism, existing studies often use traditional modules such as Squeeze-and-Excitation (SE) [20] and a Convolutional Block Attention Module (CBAM) [21], whose 3 × 3 kernel receptive field is limited, and the computational complexity is high. The LSKA module in this paper uses a separable large kernel strategy to expand the effective receptive field while reducing the computational complexity by 42%, which is more suitable for low-power consumption scenarios of drones. In summary, existing models are mostly limited to single-module optimization and have not formed a collaborative system of “architecture-fusion-attention”. This paper achieves a comprehensive improvement in detection accuracy, model lightweight, and real-time performance through multi-dimensional innovation, while maintaining similar optimization goals.

This paper is structured as follows: Section 2 elaborates on the technical solution of the proposed model, including the construction of a dual-branch framework, a semantic detail injection module, a bidirectional adaptive feature fusion mechanism, and the principle of separable large-core attention. Section 3 verifies the model effect from the dimensions of ablation analysis, comparative experiments, and generalization performance through experiments on the VisDrone2019 dataset and the RSOD dataset, and illustrates the detection advantages in combination with visualization results. Finally, Section 4 summarizes the research contribution, clarifies the accuracy and lightweight advantages of the model in drone small target detection, and looks forward to future optimization directions.

## 2. Methods

### 2.1. Overall Structure of the Model

**Figure 2:**
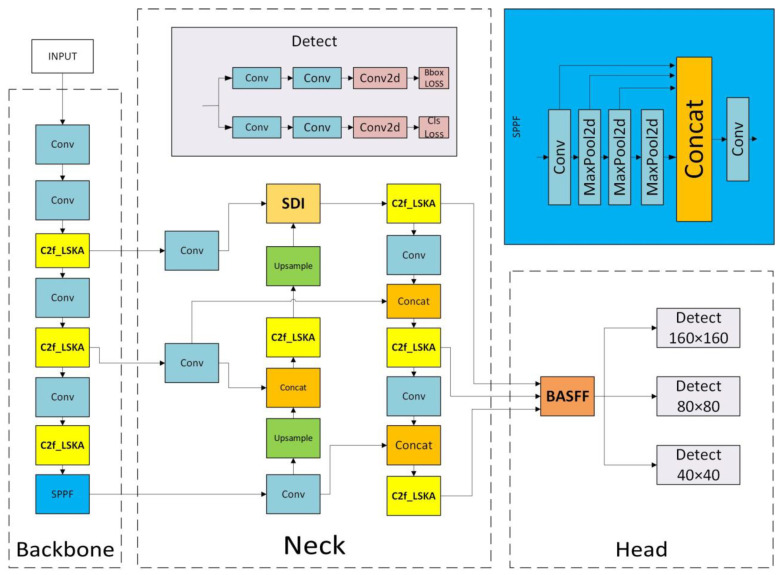
Target detection structure diagram.

The model, shown in Figure 2, is designed to improve the ability to capture small targets; a small target detection layer is introduced, and the P5 layer, which has limited effect on small targets, is removed, thereby reducing the number of model parameters. In the feature pyramid network of the YOLO model, the features of the layers P2 to P5 play different roles in target detection. Specifically, P2 to P5 correspond to feature maps that are downsampled 4 times, 8 times, 16 times, and 32 times, respectively, and the corresponding layers are the convolutional layers that generate the feature maps. Among them, the P2 layer, as a shallow feature layer, retains rich spatial details and low-level features, which are crucial for capturing small targets with fine-grained structures. The P5 layer is the deepest feature layer with low spatial resolution, but it accumulates strong semantic information through multiple downsamplings, so it mainly relies on high-level contextual features to detect large targets. As the highest layer of the feature pyramid, P5 loses spatial details due to deep downsampling, and the detection effect on small targets is poor. To retain more original details, a new dual-branch model framework of the backbone and neck, and the backbone and head are constructed with a convolution (Conv) as a bridge. At the same time, to make up for the insufficient capability of the feature pyramid network, the Semantic Detail Injection (SDI) [22] module is introduced to replace the feature fusion of the neck of the P2 layer in YOLOv8, thereby improving the accuracy of small target detection and the adaptability of the model to complex scenes. In response to the problem of inconsistent feature fusion in the YOLOv8 detection head, the Adaptive Spatial Feature Fusion (ASFF) [23] module is combined with the Bidirectional Feature Pyramid Network (BiFPN) to propose a lighter Bidirectional Adaptive Spatial Feature Fusion (BASFF) module. Finally, the Large Separable Kernel Attention (LSKA) [24] can automatically learn the importance weights of different features and focus on the features with more semantic information. This paper uses this attention to optimize the C2f module to form the C2f_LSKA module, which reduces missed detections and false detections, improves computational efficiency, and enhances model robustness. Based on the above series of optimizations, this paper proposes the DSBL model, where D represents the dual-branch network, S represents the SDI module, B represents the BASFF module, and L represents the LSKA module.

### 2.2. Dual-Branch Architecture

In the field of target detection, the original YOLOv8 detection head architecture consists of a small target detection head, a medium target detection head, and a large target detection head. Among them, the small target detection head is responsible for processing feature maps of 80 × 80 size, the medium target detection head corresponds to a feature map of 40 × 40, and the large target detection head is for a feature map size of 20 × 20. However, in actual applications, it is found that the model has certain limitations in the detection of small targets. In order to effectively improve this situation, researchers have tried to add a P2 layer small target detection head to the model.

**Figure 3:**
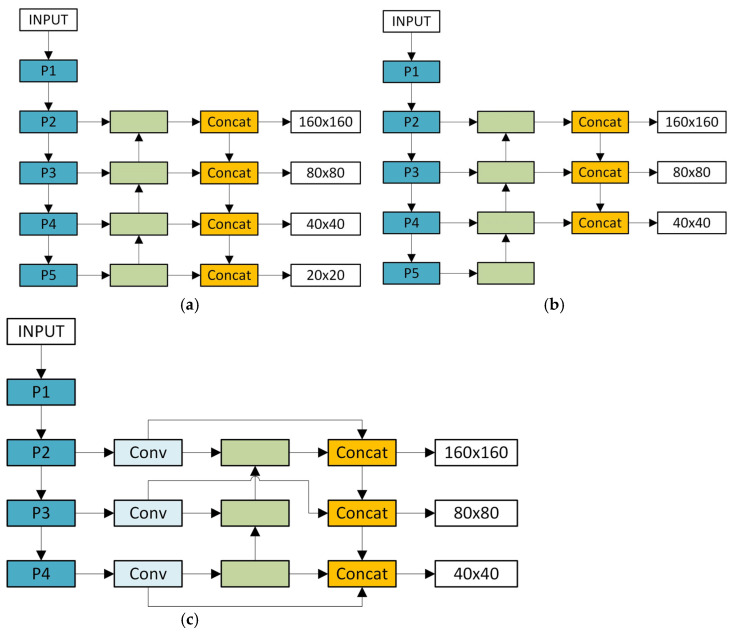
Simplified framework diagram: (**a**) four sensor head versions; (**b**) triple head version with micro sensor head; (**c**) dual-branch architecture.

At present, the relevant research on adding a detection head to the P2 layer has mainly derived two representative versions. One is the four-head version of P2, P3, P4, and P5, and the other is the three-head version of P2, P3, and P4. The structures of these two versions are intuitively presented in Figure 3a,b. The four-head version shown in Figure 3a adds an upsampling on the original basis to enable the detection head to smoothly fuse the P2 layer features through the neck. After adding the P2 layer detection head, the detection heads at each level can extract information from feature maps of different scales, and the accuracy in small target detection is much improved compared to the original model. The three-head version shown in Figure 3b removes the P5 layer, allowing the model to focus on feature extraction and target detection in the P2, P3, and P4 layers, which simplifies the model structure to a certain extent.

Inspired by these two model structures, a new dual-branch framework module is proposed. We remove the P5 layer, which has a limited effect on small targets, and focus the model on the P2, P3, and P4 layers. At the same time, we perform Conv convolution operations on the input feature map before feature fusion to further explore more advanced and abstract features. At the same time, we build a dual-branch architecture that interconnects the backbone network with the neck network and the backbone network with the head network. This framework changes the expression form of features and enhances the diversity of features, which helps to improve the model’s ability to detect targets. This dual-branch structure makes the entire detection process more focused on retaining high-resolution features. High-resolution features can retain more image details. These details are crucial and can greatly enhance the model’s ability to capture small targets. The dual-branch framework structure diagram is simplified in Figure 3c.

### 2.3. Semantics and Detail Injection

**Figure 4:**
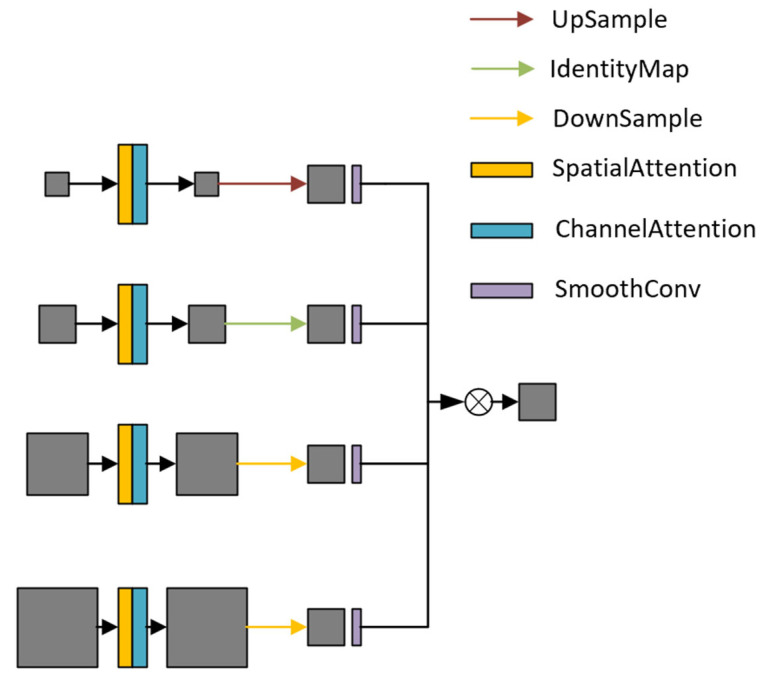
SDI schematic.

In the U-NetV2 [22] model, the SDI module integrates the semantic information of high-level features and the details of low-level features into the corresponding feature maps of each layer through an innovative fusion method, significantly enhancing the feature expression. The operation mechanism of the SDI module is as follows: First, the features of each level generated by the encoder are processed using the spatial and channel attention mechanisms. Then, the number of channels is reduced using a 1 × 1 convolution. Next, the size of the feature maps of other levels is adjusted to make their resolution consistent through a series of operations, including adaptive average pooling downsampling, identity mapping, and bilinear interpolation upsampling. The formula for adjusting the size of the feature map is as follows:

(1) $$f_{ij}^{2}=\left\{\begin{array}{c} D\left(f_{j}^{1},\left(H_{i},W_{i}\right)\right)\;j<i\\ I\left(f_{j}^{1}\right)\;j=i\\ U\left(f_{j}^{1},\left(H_{i},W_{i}\right)\right)\;j>i \end{array}\right.$$

where ***D***, ***I***, and ***U*** represent adaptive average pooling, identity mapping, and bilinear interpolation, respectively, and $H_{i}$ and $W_{i}$ represent the width and height of the feature, respectively. Adjust $f_{j}^{1}$ to the resolution $H_{i}$, $W_{i}$, and 1 ≤ *i* + *j* ≤ *M*. After completing the resolution adjustment, the SDI module applies a 3 × 3 convolution to all adjusted feature maps to smooth the feature maps and reduce noise. Subsequently, all feature maps with the same resolution are fused through element-level Hadamard products to further enrich the feature expression, so that it can more effectively capture the key information of the target area. The SDI principle is shown in Figure 4.

Introducing the SDI module into the YOLOv8 model to replace the Concat operation of the P2 layer neck network can effectively enhance the capabilities of the feature pyramid network. Compared with the simple feature concatenation of the traditional Concat module, SDI achieves the semantic-detail deep coupling of cross-layer features through a dual-stream information fusion mechanism, which effectively solves the information attenuation problem in the cross-layer feature fusion of the traditional pyramid network. Secondly, for the problem of small target detection, SDI’s unique detail retention ability can maintain high-frequency texture features during upsampling, improve the accuracy of small target detection, and enhance the adaptability of the detection model to complex scenes.

### 2.4. Bidirectional Adaptive Spatial Feature Fusion

**Figure 5:**
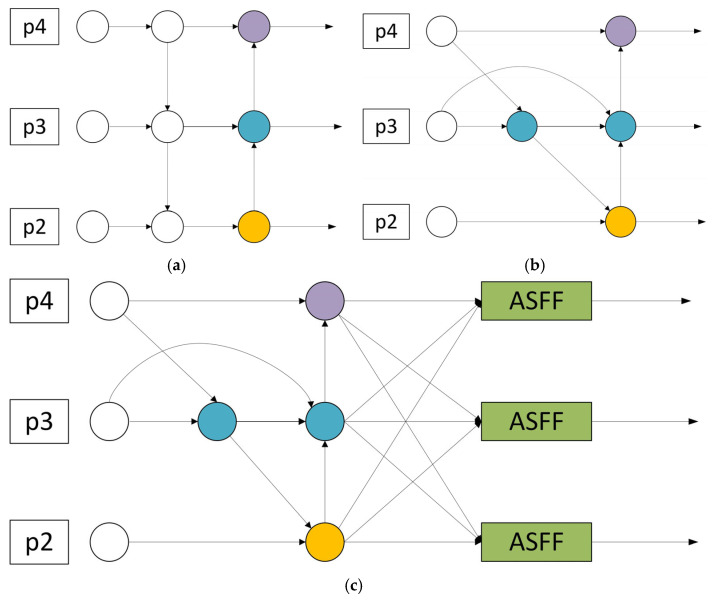
BASFF evolution: (**a**) PANet structure diagram; (**b**) BiFPN structure diagram; (**c**) BASFF structure diagram.

PANet only fuses features through a top-down path and lacks reverse detail supplementation. It enhances key areas through complex attention, but the computational cost is high, and the flexibility of feature fusion is insufficient. Therefore, inspired by the BiFPN structure, we remove the nodes with smaller contributions in the original structure and combine the top-down and bottom-up bidirectional paths to achieve a lightweight effect. However, since the features of all layers cannot be fully utilized, to solve this problem, we further introduce a feature fusion module from the ASFF module to form a new lightweight adaptive fusion module, BASFF, as shown in Figure 5. ASFF enables the network to learn how to spatially filter and combine features of different levels through feature rescaling and adaptive fusion, thereby improving the scale invariance of features and optimizing feature fusion effects. Its core advantage lies in the adaptive weight adjustment mechanism, which can effectively coordinate the problem of inconsistent gradients. The formula for fusing feature maps of different levels is as follows:

(2) $$y_{ij}^{l}=\alpha_{ij}^{l}\cdot x_{ij}^{1-l}+\beta_{ij}^{l}\cdot x_{ij}^{2-l}+\gamma_{ij}^{l}\cdot x_{ij}^{3-l}$$

where $\;y_{ij}^{l}$ represents the (*i*,*j*)th vector in the output feature map $y^{l}$, $\alpha_{ij}^{l}$, $\beta_{ij}^{l}$, and $\gamma_{ij}^{l}$ are the spatial importance weights of the feature maps of three different levels to level l, which are defined by the softmax function, and $x_{ij}^{n-l}$ represents the feature vector at the (*i*,*j*) position of the feature map from the nth layer to the lth layer.

After the BASFF module is added to the detection head, the model can adaptively learn the spatial fusion weights of features at different levels and dynamically adjust the contribution ratio of features for targets of different scales. BASFF can enhance the low-level high-resolution features of small object detection according to the size and position information of the object, and use high-level semantic features to improve the stability of large object detection, reduce feature conflicts, and improve detection accuracy. For small target detection, BASFF enhances the feature expression of small targets by fusing high-level semantic information and low-level detail information, making the detection head more sensitive to small targets, thereby improving the detection accuracy and recall rate of small targets, and the new structure is more reasonable and lightweight.

### 2.5. Large Separable Kernel Attention and C2f_LSKA

**Figure 6:**
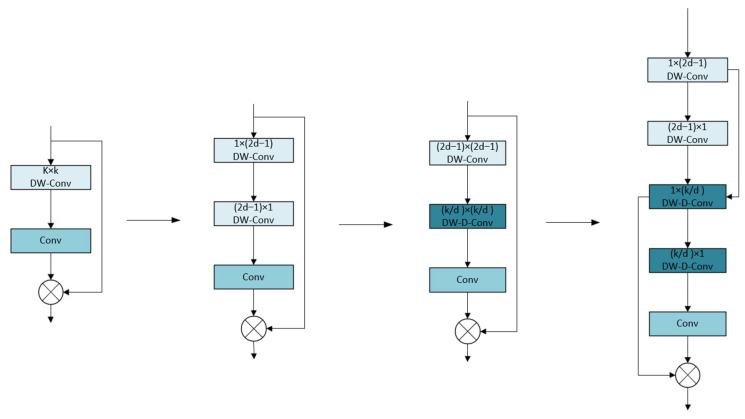
LSKA evolution diagram.

When the large kernel attention (LKA) module processes large kernel sizes, the computational cost of deep convolution is extremely high, which seriously limits the operating efficiency of the model. The LSKA module reconstructs the module architecture through the kernel decomposition strategy. LSKA shows significant advantages over traditional attention mechanisms, SE, and CBAM. First, LSKA splits the large-size 2D convolution kernel into two 1D separable kernels through the kernel decomposition strategy, which greatly reduces the computational complexity and achieves a lightweight design while ensuring model performance. Second, LSKA’s large kernel design can capture long-distance spatial dependencies, and its effective receptive field is much larger than the 3 × 3 kernel of SE and CBAM, which can better integrate contextual information such as background textures around small targets and improve target differentiation capabilities in complex scenes. Third, LSKA dynamically adjusts the receptive field through the expansion rate d to achieve adaptive fusion of multi-scale features. In dense small target detection, its ability to integrate fine-grained details and global semantics is significantly better than SE and CBAM. Figure 6 shows the evolution of LSKA from the basic large kernel attention module.

In terms of the calculation process, the input feature map is first subjected to 1D convolution in the horizontal and vertical directions. Although the subsequent 1 × 1 convolution and Hadamard product operations are similar to the original LKA module, the overall calculation complexity is significantly reduced. It successfully achieves a significant reduction in calculation complexity and memory usage while ensuring model performance. The output formula of the LSKA module is as follows:

(3) $${\bar{Z}}^{C}=\sum_{H,W}W_{\left(2d-1\right)\times 1}^{C}*(\sum_{H,W}W_{1\times \left(2d-1\right)}^{C}*F^{C})$$

(4) $$Z^{C}=\sum_{H,W}W_{\lfloor \frac{k}{d}\rfloor \times 1}^{C}*\left(\sum_{H,W}W_{1\times \lfloor \frac{k}{d}\rfloor }^{C}*{\bar{Z}}^{C}\right)$$

(5) $$A^{C}=W_{1\times 1}*Z^{C}$$

(6) $${\bar{F}}^{C}=A^{C}⨂F^{C}$$

where *k* represents the convolution kernel size, ∗ represents convolution, ⨂ represents the Hadamard product, d represents the dilation rate, and ${\bar{Z}}^{C}$ represents the output of the first convolution stage in the LSKA module. The input feature map $F^{C}$ is first convolved with a 1D convolution kernel of size 1 × (2d − 1) and then convolved with a 1D convolution kernel of size (2d − 1) × 1 to obtain ${\bar{Z}}^{C}$. ${\bar{Z}}^{C}$ is then convolved with two 1D convolution kernels of size $\left\lfloor \frac{k}{d}\right\rfloor$ × 1 and 1 × $\left\lfloor \frac{k}{d}\right\rfloor$ to obtain $Z^{C}$. $Z^{C}$ is then convolved with a convolution kernel of (1 × 1) to obtain the attention map $A^{C}$. Finally, the attention map $A^{C}$ is Hadamard-produced with the original input feature map $F^{C}$ to obtain the final output ${\bar{F}}^{C}$.

**Figure 7:**
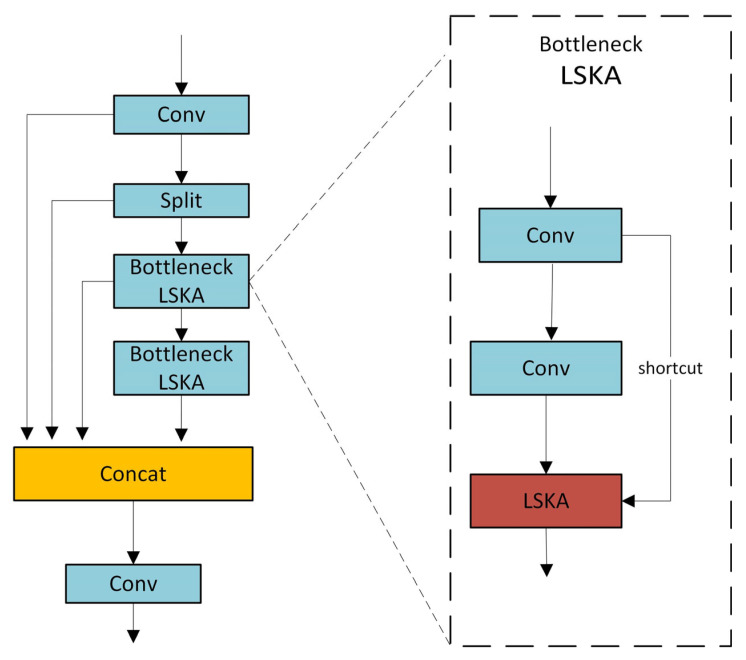
C2f_LSKA structure diagram.

We integrate the LSKA module into the C2f module to form a new module C2f_LSKA, the structure of which is shown in Figure 7, which further enhances the performance of the model in the target detection task. The C2f module adopts an advanced cross-stage local network structure and has a strong feature integration capability. It can effectively integrate feature information at different levels during the target detection process. The embedded LSKA module provides it with a richer and more comprehensive feature representation. In the target detection task of complex scenes, the accurate recognition of small targets depends on shallow detail features, while large targets require deeper semantic feature support. After the C2f module integrates these different levels of features, the LSKA module can focus on the target area more accurately, significantly improve the detection accuracy of targets of different scales, and effectively reduce missed detection and false detection.

In addition, the C2f module itself has the advantages of reducing redundant calculations and reducing the model calculation cost. While maintaining the model’s performance, it can reduce the number of parameters and the amount of calculation. When the LSKA module is integrated with it, it can reduce the computational complexity by decomposing the large kernel, and the two work together to further improve the computational efficiency of the model. This optimization enables the model to quickly process image data and achieve real-time target detection in resource-constrained environments, such as drone systems. Even with limited hardware resources, the fused model can efficiently process video images and quickly detect target objects, showing strong practicality.

The effective fusion of features at different levels by the C2f module gives the model strong adaptability to various targets and scenes. The addition of the LSKA module, with its good adaptability to long-distance dependence and spatial channels, further enhances the model’s ability to extract target features. When faced with different data sets or complex actual scenes, such as images with changing lighting conditions and complex backgrounds, the C2f model incorporating LSKA can effectively reduce overfitting and significantly improve the robustness of the model, thereby stably detecting targets and greatly enhancing the practical value of the model.

## 3. Results

### 3.1. Dataset

This paper uses VisDrone2019 [25], captured by the AISKYEYE team of the Machine Learning and Data Mining Laboratory of Tianjin University. This dataset covers a wide range of scenes with different background complexities, from sparse to crowded. The images are taken under different lighting intensities and weather conditions, including daytime, night, cloudy, and foggy environments. It contains a total of 10,209 static images, annotated with ten target categories, such as cars, trucks, and pedestrians. In particular, due to the high altitude of drones, there are a large number of small targets in the dataset, and dense small targets in busy street scenes often overlap and occlude, which further increases the difficulty of detection. The dataset is divided according to the official standard, with 6471 training sets, 548 validation sets, and 1610 test sets, with a division ratio of about 12:1:3.

### 3.2. Evaluation Indicators

In order to evaluate the performance of the model and whether it can be built on a resource-limited platform such as a drone, the following six indicators are selected to evaluate the performance, including precision (*P*), recall (*R*), mean average precision (*mAP*), Params, model size, and Frames Per Second (FPS).

Precision refers to the proportion of samples that are truly positive among all samples predicted by the model to be positive, reflecting the accuracy of the model’s prediction results. The calculation formula is as follows:

(7) $$P=\frac{TP}{TP+FP}$$

where *TP* stands for true positive, and FP stands for false positive.

The recall rate refers to the proportion of samples that the model correctly predicts to be positive. It measures the model’s ability to capture positive samples, that is, how many actual positive samples the model can find. The calculation formula is as follows:

(8) $$R=\frac{TP}{TP+FN}$$

where *FN* represents false negative examples.

The mean average precision is calculated at different recall levels, and the average of these accuracies is obtained, which can more comprehensively reflect the performance of the model. The higher the *mAP* value, the better the detection performance of the model. The calculation formula is as follows:

(9) $$mAP=\frac{1}{N}\sum_{i}^{N}\int_{0}^{1}P\left(R\right)dR$$

Params refers to the number of all learnable parameters in the model, including the weights of the convolutional layer, the bias term, and the weights of the fully connected layer. The fewer the parameters, the lighter the model; the model size refers to the size of the space occupied by the weight file on the storage device. A smaller model size is conducive to the deployment and transmission of the model, especially on resource-constrained devices, where model size is an important consideration. The frame rate per second indicates the number of image frames that the model can process per second. FPS reflects the inference speed of the model, that is, how fast the model detects objects in the input image and outputs the results, and is an important indicator for evaluating the real-time performance of the model.

### 3.3. Experimental Environment and Configuration

The experiment was built on a computer platform with Windows 10. The graphics card model is an NVIDIA GeForce RTX 4070 (12282MiB), the CUDA version is 12.4, the Python version is 3.8.2, and the Torch version is 1.12.0. For the key hyperparameters of the YOLOv8 framework, we performed five-fold cross-validation on the initial learning rate lr, momentum, and weight_decay, and the parameter search ranges are lr ∈ [0.01, 0.001, 0.0001], momentum ∈ [0.9, 0.937, 0.95], weight_decay ∈ [0.0001, 0.0005, 0.001]. After the VisDrone2019 dataset is divided, each fold is trained independently for 30 rounds, with mAP50 as the core evaluation indicator. This method ensures the generalization of parameter optimization through multi-fold verification.

**Figure 8:**
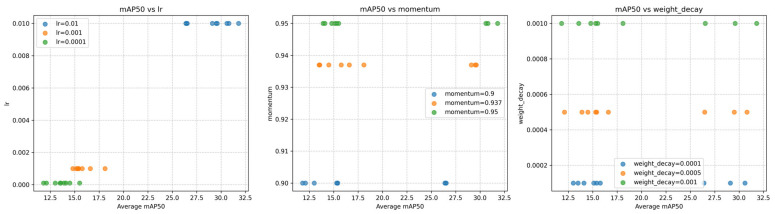
Relationship diagram of three hyperparameters and mAP50.

The experimental results are shown in Figure 8, which reflects the relationship between the three types of hyperparameters and the average precision mAP50 from left to right. The data shows that the model works best when lr = 0.01, momentum = 0.95, and weight_decay = 0.001, thus ensuring the reliability and comparability of the results. Table 1 lists the final experimental parameter settings in detail. The parameters not mentioned are all the official default values of YOLOv8.

### 3.4. Parameter Selection of C2f_LSKA

**Figure 9:**
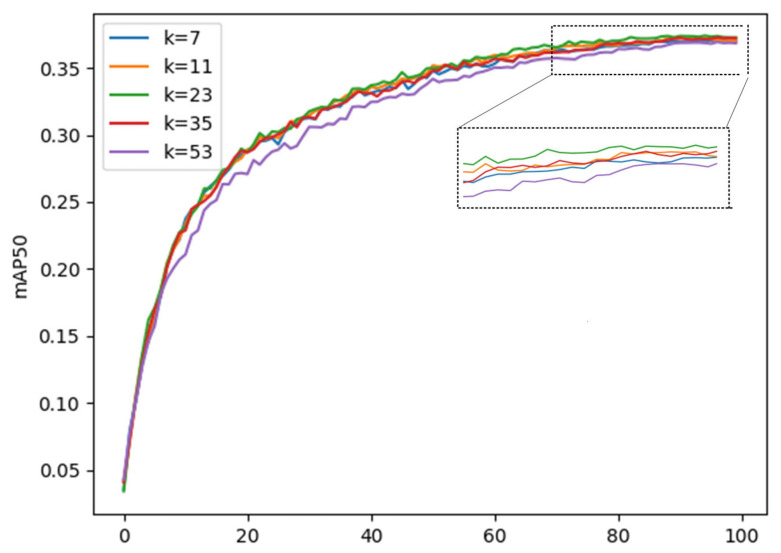
Experimental graphs with different convolution kernel sizes.

As mentioned above, the performance of the C2f_LSKA module is affected by the convolution kernel size k value. In order to make the module better adapted to the small target detection task, we conducted experiments on the YOLOv8 initial model for the k value, and the value range was set between 7 and 53. The experimental results are shown in the Figure 9, which clearly shows the fluctuation trend of the model mean average precision mAP50 as the k value changes. It can be seen from the figure that when k is from 7 to 23, the mean average precision mAP50 increases with the increase in k value; when k is from 23 to 53, the mean average precision mAP50 decreases with the increase in k value. At the same time, the increase in k value is accompanied by an increase in parameters. Considering comprehensively, when the k value is selected as 23, the comprehensive effect of the model is most ideal.

### 3.5. Ablation Experiment

**Figure 10:**
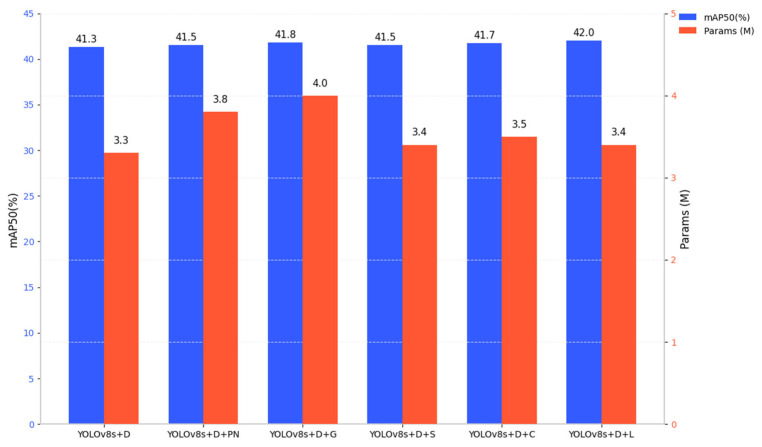
Comparison of mAP50 and number of parameters of different attention mechanisms.

In order to verify the effectiveness of the LSKA attention mechanism on the model, this paper adds different attention mechanisms at the same position on the basis of the dual-branch structure for experimental comparison. The experimental results are shown in Table 2 and the supporting bar graph is shown in Figure 10. As can be seen from Table 2, after the introduction of the LSKA attention mechanism, the model only adds a small number of parameters, and the mAP50 value reaches its highest. Among them, the accuracy of the ParNet (PN) [26], GAM (G) [27], SE (S), and CBAM (C) attention mechanisms are higher than the baseline model, but not higher than LSKA, and the number of parameters of GAM increases the most. Therefore, this paper chooses the LSKA attention mechanism, which can help the network extract important feature information, reduce the interference of complex information, and effectively improve network performance.

To explore the indispensability of different parts in the model architecture, we conducted an ablation experiment on the model. Based on the original YOLOv8s framework, we gradually added module components on the VisDrone2019 dataset to verify the performance. The ablation experiment results are shown in Table 3.

**Figure 11:**
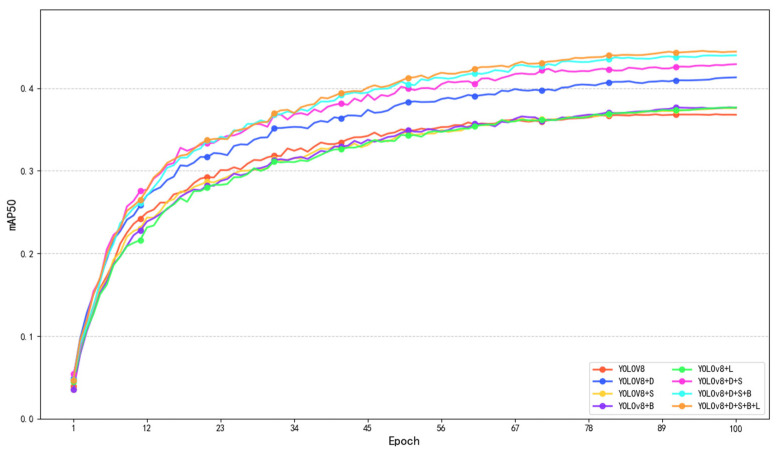
Ablation experiment mAP change curve.

From the table, we can see that when adding a single module to the original YOLOv8s, each mAP50 is improved. Among them, the D module has the largest improvement, which is 12.2%, and the number of parameters has also decreased the most, which is 7.8M, a decrease of 70.3% of the original parameters. The S module mAP50 increased by 2.2%, but the number of parameters increased by 0.8M. The B module mAP50 increased by 2.5%, and the number of parameters increased by 5.4M; the L module mAP50 increased by 2.2%, but the number of parameters increased by 0.3M. From the addition of a single model, we can conclude that model D has a dual-branch framework, which not only greatly reduces the total number of model parameters and algorithm size, but also significantly improves mAP50, which is conducive to the configuration of the model on drones. The mAP50 of other single modules has been improved to a greater or lesser extent, but is accompanied by an increase in the number of model parameters. However, from Figure 11, we can clearly see that each module plays an important role in small target detection. We further add module S on the basis of module D, and the mAP50 increases by 3.8%, an increase of 16.6% relative to the basic model, and the number of parameters decreases by 67.6% relative to the basic model, indicating that the addition of the SDI module improves the model’s perception and recognition capabilities of small target areas. We add module B on this basis again, and the mAP50 increases by 2.6%, an increase of 19.6% relative to the basic model, and the number of parameters decreases by 62.2% relative to the basic model, indicating that the addition of the BASFF module improves the scale invariance of the features while avoiding interference with the target gradient. Finally, the model DSBL we proposed has a 20.9% increase in mAP50 and a 23.7% increase in mAP50–95 relative to the original model, a 61.3% decrease in total parameters, and a 57.5% decrease in algorithm size, which shows that each module of the model cooperates with each other to perform well in the field of small targets in drone aerial photography.

### 3.6. Comparative Experiment

#### 3.6.1. Comparison with the YOLOv8 Network

In order to evaluate the performance improvement of the DSBL model compared to YOLOv8, this section analyzes the confusion matrix and the precision–recall (P-R) curve. The confusion matrix uses rows and columns to represent the true category and the predicted category. The color depth reflects the sample ratio, the diagonal line represents the correct classification, and the non-diagonal line reflects the degree of category confusion, which intuitively shows the model’s ability to distinguish category boundaries. The P-R curve predicts the proportion of true positive samples in positive samples and the proportion of true positive samples that are correctly detected by dynamically balancing precision and recall, and uses the area under the curve (AP) and multi-category average mAP to quantify the detection accuracy. The closer the curve is to the upper right corner, the better the performance.

**Figure 12:**
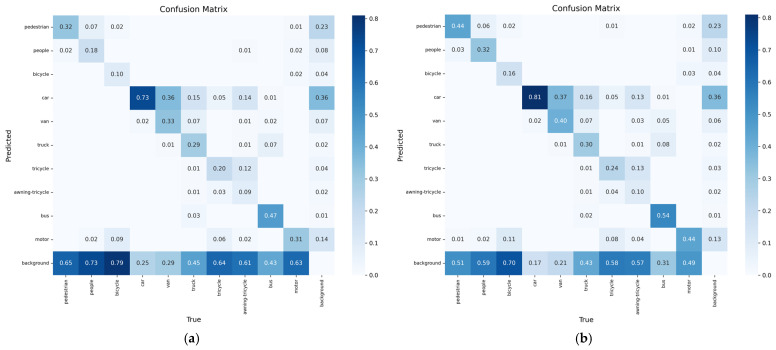
Confusion matrix: (**a**) YOLOv8; (**b**) DSBL.

The confusion matrix is shown in Figure 12. The confusion matrix clearly shows the performance difference between YOLOv8 and DSBL. In the confusion matrix of YOLOv8, cross-category misclassification is significant, “pedestrian” is often misclassified as “people”, and there is obvious cross-category confusion between “car” and “van”. Such errors reflect the model’s insufficient ability to distinguish objects with similar features and semantic similarity, the scattered distribution of diagonal elements, and the low correct classification rate. In contrast, the confusion matrix of DSBL shows a more concentrated diagonal distribution, the correct classification rate of pedestrians is significantly improved, and the misclassification frequency of complex structural objects such as “bicycle” and “awning-tricycle” is reduced. This improvement is due to the newly added feature fusion module and LSKA attention mechanism. The former enriches the semantic representation by aggregating cross-scale complementary features, and the latter adaptively focuses on category-specific discriminative details. The two work together to enhance the model’s ability to distinguish different categories.

**Figure 13:**
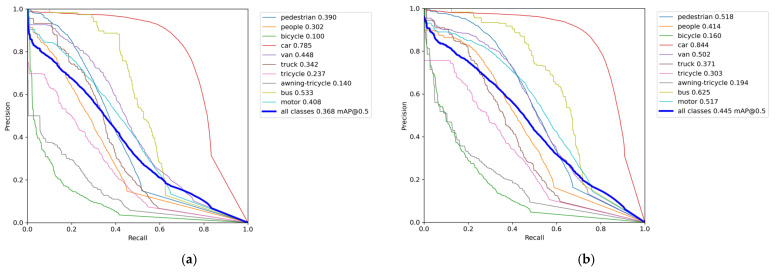
P-R Curve: (**a**) YOLOV8; (**b**) DSBL.

The precision–recall curve is shown in Figure 13. The overall curve of DSBL is closer to the upper left corner of the coordinate system. It has significant advantages in small target categories such as pedestrians, people, bicycles, and global detection. The AP of pedestrians increased from 0.390 to 0.518, an increase of 32.8%. The AP of people increased from 0.302 to 0.414, and the AP of bicycles increased from 0.100 to 0.160, an increase of 60%. The performance improvement is due to the optimization of feature representation by DSBL. The newly added feature fusion module strengthens the complementarity of cross-scale features. The LSKA attention accurately captures the target details, so that the model still maintains high precision in the low recall stage and reduces false detection in the high recall stage, and finally achieves a breakthrough in all-category detection, especially highlighting the improvement of small target detection capabilities.

#### 3.6.2. Comparison with Mainstream Models

To further verify the reliability of the model, a comparative experiment was conducted on the VisDrone2019 dataset for the proposed model and the mainstream target detection network model. To evaluate the superiority of the model compared with the current mainstream target detection algorithm, the specific experimental results are shown in Table 4.

**Figure 14:**
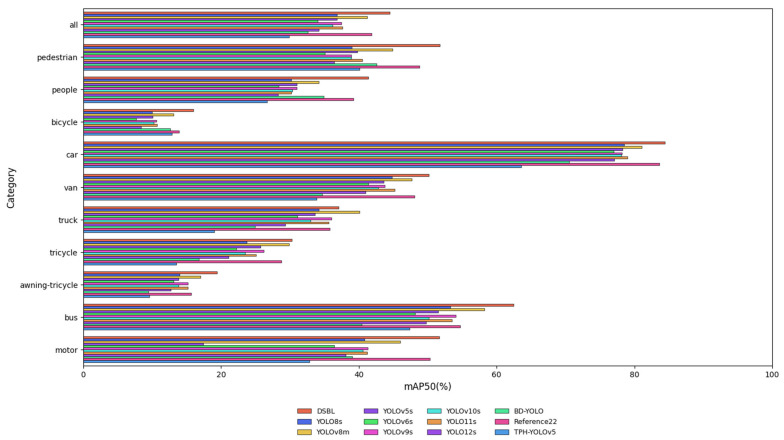
Comparison of mAP50 of each category of detection model.

The BSAL model shows significant comprehensive performance advantages in target detection tasks. In public benchmark tests, its mAP50 and mAP50–95 reached 43.6% and 26.4% respectively, which is better than the mainstream YOLO series and improved models. In terms of model parameter volume and storage usage, BSAL only requires 4.3 M parameters and 9.1 MB volume, while achieving a real-time inference speed of 78.6 FPS. As can be seen from Figure 14, the DSBL model has a significantly higher detection accuracy than baseline models, such as YOLOv8s and YOLOv12s, for medium and large targets, such as cars and buses, as well as small and dense targets, such as pedestrians and bicycles, fully demonstrating its robustness to multi-scale targets. Experiments show that the model effectively alleviates the trade-off between accuracy and efficiency in lightweight design through structural optimization, and provides a feasible solution for mobile and edge computing scenarios that takes into account high accuracy, low resource consumption, and real-time performance.

### 3.7. Generalization Experiment

**Figure 15:**
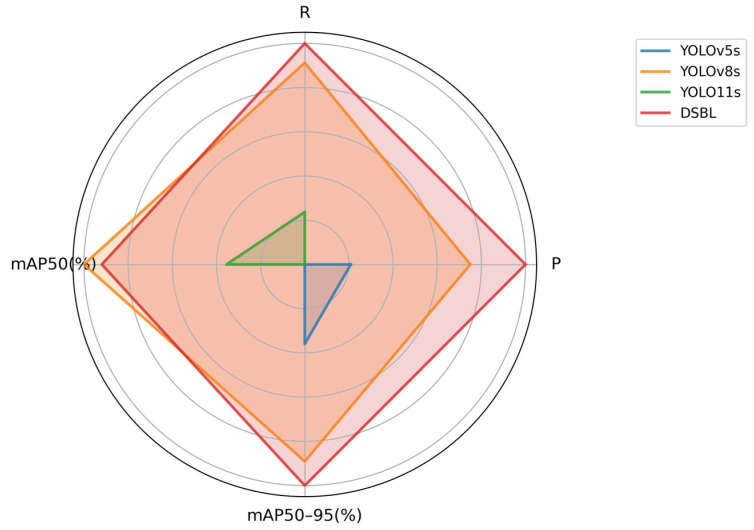
Model comprehensive performance comparison radar chart.

To avoid the accidental performance of DSBL on the dataset, we further verified the model performance on the RSOD dataset [31], and the results are shown in Table 5 and Figure 15. From the table, we can see that although DSBL on the RSOD dataset is 0.2 percentage points lower than the original model in mAP50, it has a lot of improvements in precision, recall, and mAP50–95, which shows that the model can also achieve effective improvement in the small target detection task of remote sensing images.

### 3.8. Visual Analytics

**Figure 16:**
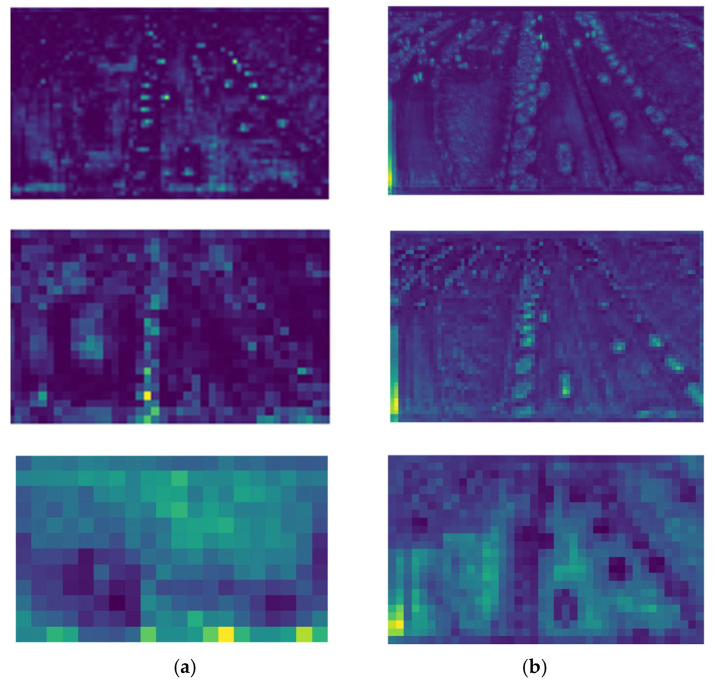
Feature Map: (**a**) YOLOv8; (**b**) DSBL.

Figure 16 shows the feature maps of the original YOLOv8s and DSBL model detection head inputs, respectively. By comparison, the distinction between the target and the background in the feature map of YOLOv8s is relatively vague. There are many environmental interference factors, and the feature distribution is scattered, which may cause the detection to be affected by the surrounding environment, and it is difficult to accurately focus on the target. The feature map of the DSBL model is obviously more concentrated on the target itself, the target outline and key features are prominent, and the background interference is effectively suppressed. This difference stems from the C2f_LSKA module of DSBL, whose cross-stage local network structure achieves the effective fusion of features at different levels. Combined with the characteristics of the LSKAttention module, the model can accurately capture target features and reduce environmental interference. Therefore, in drone target detection, DSBL can more accurately identify targets, reduce misjudgments and misjudgments caused by environmental interference, and improve detection accuracy.

**Figure 17:**
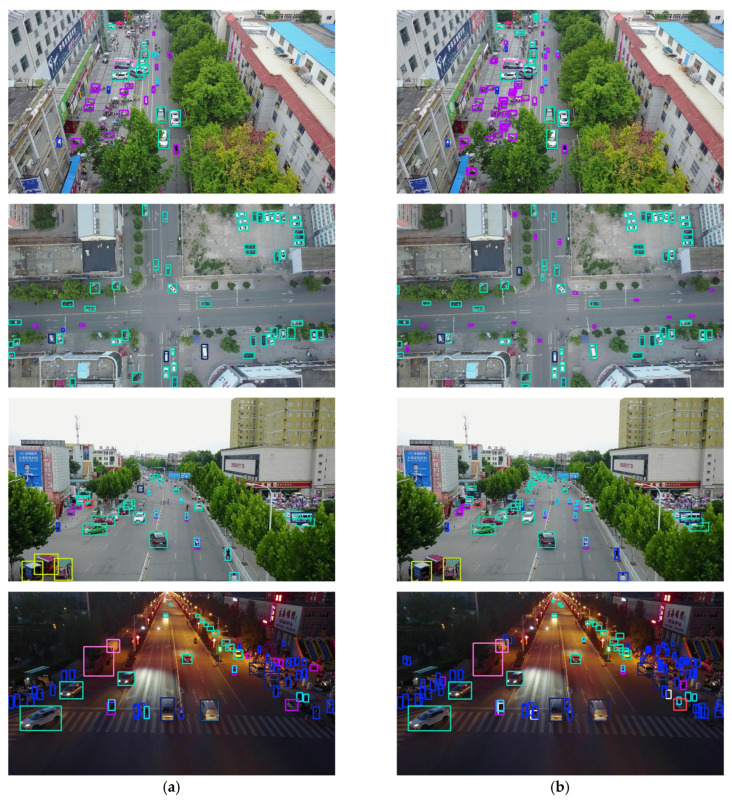
Detection images in different scenarios: (**a**) YOLOv8; (**b**) DSBL.

Figure 17 shows the detection images of the original YOLOv8s and DSBL models in different scenarios. After comparison, DSBL has significant advantages in a variety of complex scenarios. In the scenarios of looking down and looking at an angle, the detection frame of DSBL fits the shape and position of the target better, while YOLOv8s is prone to detection frame offset or scale estimation deviation when looking at an angle. In terms of light conditions, DSBL maintains good detection results both during the day and at night. Especially at night, it can still clearly identify the target and accurately locate it, while YOLOv8s is prone to missed detection or misjudgment due to insufficient light. In terms of target distribution, DSBL can better distinguish adjacent targets in dense scenes, reduce overlapping and confusion of detection frames, and accurately identify each target in sparse scenes to avoid misjudgment caused by background interference. In summary, DSBL can more accurately identify targets in all kinds of scenarios, and the detection frame is more accurately located, which effectively improves the accuracy and reliability of detection, and has obvious advantages over the original YOLOv8s model.

### 3.9. Practical Scenario Validation

To further verify the applicability of the proposed model in actual UAV scenarios, two types of validation experiments are conducted: temporary dataset testing and field flight simulation, which aim to evaluate the performance of the model in complex real-world environments.

#### 3.9.1. Temporary Dataset Construction and Results

We randomly selected 300 images from two public UAV aerial datasets, VisDrone-2021val and CODrone, and combined them with 349 images captured by our team to construct a temporary test set containing 949 images. This dataset covers multiple scenarios such as urban areas, rural regions, and roadways, and includes UAV-specific environmental interferences such as flight jitter, high-altitude perspective distortion, and cluttered backgrounds, which are highly consistent with actual application scenarios.

**Figure 18:**
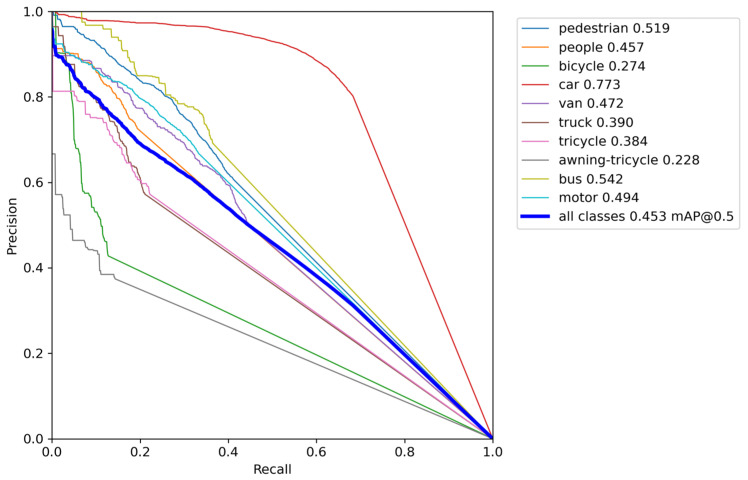
P-R curve of the model on the temporary dataset.

The trained model weights were directly used for testing on this temporary dataset. Experimental results showed that among 949 images containing 56,454 targets, the model achieved a precision of 59.3%, a recall of 31.5%, an mAP50 of 45.3%, an mAP50–95 of 28.9%, and an FPS of 158.4. Compared with performance on the VisDrone2019 dataset, where the mAP50 was 44.5% and FPS was 78.6, the slight increase in mAP50 (0.8%) may be attributed to the diverse scene samples in the temporary dataset, verifying the model’s adaptability to new environments. The significant improvement in FPS (158.4 vs. 78.6) is due to the absence of complex data augmentation in the temporary dataset, which is more consistent with real-time UAV deployment requirements. In subcategories, the model performed well in detecting vehicle targets and pedestrians, and also exhibited strong recognition capabilities for small and dense targets commonly seen in UAV scenarios. The precision–recall curve of the model on the temporary dataset is shown in Figure 18.

#### 3.9.2. Field Flight Test and Simulation

**Figure 19:**
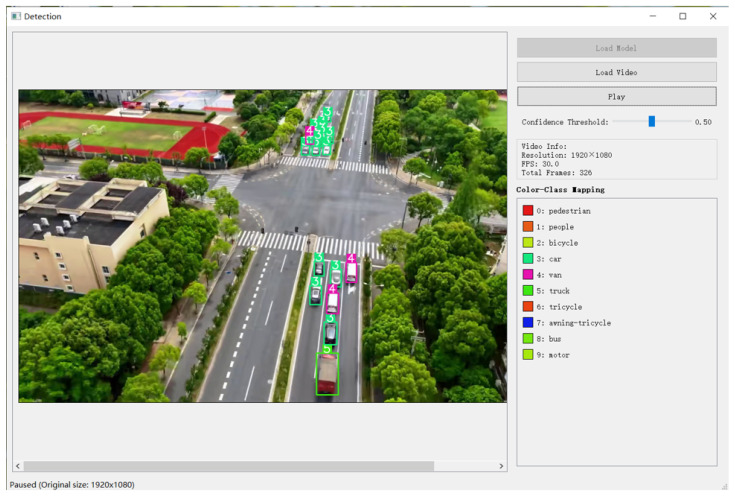
Detection interface diagram.

To further verify the model’s adaptability to actual flight scenarios, we conducted field UAV flight tests in urban blocks, recorded real-time images during the flight without additional processing, and processed and detected these recorded UAV images on a computer. The detection interface is shown in Figure 19, which displays key information such as 1920 × 1080 resolution, 30.0 FPS frame rate, 326 total frames, and color-class mapping for target categories, such as pedestrian and car.

The model showed stable real-time prediction performance for dynamic targets and maintained a smooth detection frame rate of 30 FPS, meeting real-time requirements of practical applications.

In summary, both experiments confirm that the model maintains high detection accuracy and real-time performance in actual drone scenarios. The dual-branch architecture and LSKA module effectively suppress the interference of complex backgrounds, providing potential for its application in drone deployment.

## 4. Conclusions

This study proposes a dual-branch lightweight model based on YOLOv8s to address the problem of small-target detection in drone aerial images. By building a dual-branch architecture that preserves shallow features, the number of parameters is reduced by 55%, the SDI module is integrated to enhance multi-scale feature fusion, and a lightweight adaptive feature fusion mechanism is added to the detection head. LSKA is innovatively introduced into the convolutional layer to enhance target representation, and ultimately, the mAP50 is improved by 20.9% and the mAP50–95 is improved by 23.7% in the VisDrone2019 dataset, and the model size is reduced by 57.5%, significantly improving the adaptability of drone-side deployment. However, the model still has limitations in practical applications: In temporary data sets under actual scenarios, for extremely small targets, due to the insufficient ability of the C2f_LSKA module to capture extremely low pixel features, the detection stability is lower than that of ordinary small targets, and the missed detection rate is significantly higher than that of small and medium-sized targets. At the same time, in scenarios with dense occlusions such as overlapping vehicles or pedestrians on busy streets, some overlapping targets have detection frame positioning deviations, indicating that the feature decoupling capability of the existing feature fusion mechanism in extremely dense scenarios still needs to be enhanced.

Future research will focus on the following directions: First, to optimize the computational graph and quantize the model for embedded deployment needs, and verify its real-time reasoning stability on ARM architecture processors. Second, to build a drone test platform, collect self-built data sets including dynamic perspectives and complex environments, and test the robustness of the model to motion blur and perspective distortion; Third, to introduce Transformer position encoding to achieve feature decoupling of dense scenes, and integrate infrared-visible light multimodal data to improve detection reliability in harsh environments, and promote the transformation of algorithms to engineering applications.

## Figures and Tables

**Table 1:** Training parameter settings.

Training Parameters	Parameter Value
epoch	100
batch	2
workers	8
Imgsz	640 × 640
lr	0.01
optimizer	SGD
weight_decay	0.001
momentum	0.95

**Table 2:** Attention mechanism ablation.

Attention Mechanism	mAP50 (%)	Params (M)
YOLOv8s + D	41.3	3.3
YOLOv8s + D + PN	41.5	3.8
YOLOv8s + D + G	41.8	4.0
YOLOv8s + D + S	41.5	3.4
YOLOv8s + D + C	41.7	3.5
YOLOv8 s + D + L	42	3.4

**Table 3:** Ablation experiments of various improved modules.

Yolov8s	D	S	B	L	mAP50 (%)	mAP50–95 (%)	Params (M)	Algorithm Size (MB)
√					36.8	21.9	11.1	21.4
√	√				41.3	25.1	3.3	6.59
√		√			37.6	22.4	11.9	23.1
√			√		37.7	22.6	16.5	33.5
√				√	37.6	22.3	11.4	22.0
√	√	√			42.9	26.0	3.6	7.01
√	√	√	√		44.0	26.8	4.2	8.9
√	√	√	√	√	44.5	27.1	4.3	9.1

Note: The corresponding “√” in the table indicates that the module is introduced into the framework.

**Table 4:** Comparative experimental results.

Model	mAP50 (%)	mAP50–95 (%)	Params (M)	Algorithm Size (MB)	FPS
YOLOv5s	36.8	21.8	9.1	17.7	99.3
YOLOv6s	34.0	20.2	16.3	32.8	60.1
YOLOv8s	36.8	21.9	11.1	21.4	99.0
YOLOv8m	41.2	25.3	25.9	49.6	48.5
YOLOv9s	37.5	22.4	7.2	14.5	70.5
YOLOv10s	36.2	21.7	8.0	15.7	91.2
YOLO11s	37.6	22.4	10.7	21.8	93.4
YOLO12s	34.2	20.2	9.1	18.1	90.6
Reference [28]	41.9	25.3	5.3	10.9	34.4
BD-YOLO [29]	32.6	17.9	11.1	22.5	-
TPH-YOLO [30]	29.9	16.9	8.4	17.8	-
DSBL	44.5	27.1	4.3	9.1	78.6

**Table 5:** Comparison results on RSOD dataset.

Model	P	R	mAP50 (%)	mAP50–95 (%)
YOLOv5s	86.1	80.6	86.1	60.5
YOLOv8s	88.7	87.9	92.3	63.9
YOLO11s	85.1	82.5	88.3	58.2
DSBL	89.9	88.6	91.8	64.6

## Data Availability

The dataset VisDrone2019 can be downloaded at https://github.com/five-days/VisDrone-Dataset, and the dataset RSOD can be downloaded at https://github.com/RSIA-LIESMARS-WHU/RSOD-Dataset-. All datasets accessed on 16 May 2025.

## References

[1] Dalal N., Triggs B. (2005). Histograms of oriented gradients for human detection. Proceedings of the 2005 IEEE Computer Society Conference on Computer Vision and Pattern Recognition (CVPR’05).

[2] Lowe D.G. (2004). Distinctive image features from scale-invariant keypoints. Int. J. Comput. Vis..

[3] Ojala T., Pietikainen M., Maenpaa T. (2002). Multiresolution gray-scale and rotation invariant texture classification with local binary patterns. IEEE Trans. Pattern Anal. Mach. Intell..

[4] Chen C., Liu M.Y., Tuzel O., Xiao J. (2016). R-CNN for small object detection. Asian Conference on Computer Vision.

[5] Ren S., He K., Girshick R., Sun J. (2017). Faster R-CNN: Towards real-time object detection with region proposal networks. IEEE Trans. Pattern Anal. Mach. Intell..

[6] Lin T.Y., Dollár P., Girshick R., He K., Hariharan B., Belongie S. Feature pyramid networks for object detection. Proceedings of the IEEE Conference on Computer Vision and Pattern Recognition.

[7] Liu W., Anguelov D., Erhan D., Szegedy C., Reed S., Fu C.Y., Berg A.C. (2016). Ssd: Single shot multibox detector. Proceedings of the Computer Vision–ECCV 2016: 14th European Conference.

[8] Lin T.Y., Goyal P., Girshick R., He K., Dollár P. Focal loss for dense object detection. Proceedings of the IEEE International Conference on Computer Vision.

[9] Zeng S., Yang W., Jiao Y., Geng L., Chen X. (2024). SCA-YOLO: A new small object detection model for UAV images. Vis. Comput..

[10] Edozie E., Shuaibu A.N., John U.K., Sadiq B.O. (2025). Comprehensive review of recent developments in visual object detection based on deep learning. Artif. Intell. Rev..

[11] Zhao Y., Lv W., Xu S., Wei J., Wang G., Dang Q., Liu Y., Chen J. Detrs beat yolos on real-time object detection. Proceedings of the IEEE/CVF Conference on Computer Vision and Pattern Recognition.

[12] Munteanu D., Moina D., Zamfir C.G., Petrea Ș.M., Cristea D.S., Munteanu N. (2022). Sea mine detection framework using YOLO, SSD and EfficientDet deep learning models. Sensors.

[13] Oliveira D., Mafra S. (2024). Implementation of an Intelligent Trap for Effective Monitoring and Control of the Aedes aegypti Mosquito. Sensors.

[14] Dhou S., Alnabulsi A., Al-Ali A.R., Arshi M., Darwish F., Almaazmi S., Alameeri R. (2022). An IoT machine learning-based mobile sensors unit for visually impaired people. Sensors.

[15] Fort A., Peruzzi G., Pozzebon A. (2021). Quasi-real time remote video surveillance unit for lorawan-based image transmission. Proceedings of the 2021 IEEE International Workshop on Metrology for Industry 4.0 & IoT (MetroInd4. 0&IoT).

[16] Pronello C., Ruiz X.R.G. (2023). Evaluating the performance of video-based automated passenger counting systems in real-world conditions: A comparative study. Sensors.

[17] Zhan W., Sun C., Wang M., She J., Zhang Y., Zhang Z., Sun Y. (2022). An improved Yolov5 real-time detection method for small objects captured by UAV. Soft Comput..

[18] Gromada K., Siemiątkowska B., Stecz W., Płochocki K., Woźniak K. (2022). Real-time object detection and classification by UAV equipped with SAR. Sensors.

[19] Zunair H., Khan S., Hamza A.B. (2024). RSUD20K: A dataset for road scene understanding in autonomous driving. Proceedings of the 2024 IEEE International Conference on Image Processing (ICIP).

[20] Zhang X., Li J., Hua Z. (2022). MRSE-Net: Multiscale residuals and SE-attention network for water body segmentation from satellite images. IEEE J. Sel. Top. Appl. Earth Obs. Remote Sens..

[21] Du L., Lu Z., Li D. (2022). Broodstock breeding behaviour recognition based on Resnet50-LSTM with CBAM attention mechanism. Comput. Electron. Agric..

[22] Peng Y., Chen D.Z., Sonka M. (2025). U-net v2: Rethinking the skip connections of u-net for medical image segmentation. Proceedings of the 2025 IEEE 22nd International Symposium on Biomedical Imaging (ISBI).

[23] Qiu M., Huang L., Tang B.-H. (2022). ASFF-YOLOv5: Multielement detection method for road traffic in UAV images based on multiscale feature fusion. Remote Sens..

[24] Lau K.W., Po L.-M., Yasar A.U.R. (2024). Large separable kernel attention: Rethinking the large kernel attention design in cnn. Expert Syst. Appl..

[25] Du D., Zhu P., Wen L., Bian X., Lin H., Hu Q., Peng T., Zheng J., Wang X., Zhang Y. VisDrone-DET2019: The vision meets drone object detection in image challenge results. Proceedings of the IEEE/CVF International Conference on Computer Vision Workshops.

[26] Ham T.J., Lee Y., Seo S.H., Kim S., Choi H., Jung S.J., Lee J.W. (2021). ELSA: Hardware-software co-design for efficient, lightweight self-attention mechanism in neural networks. Proceedings of the 2021 ACM/IEEE 48th Annual International Symposium on Computer Architecture (ISCA).

[27] Xiong C., Zayed T., Abdelkader E.M. (2024). A novel YOLOv8-GAM-Wise-IoU model for automated detection of bridge surface cracks. Constr. Build. Mater..

[28] Ning T., Wu W., Zhang J. (2024). Small object detection based on YOLOv8 in UAV perspective. Pattern Anal. Appl..

[29] Wu M.J., Yun L.J., Chen Z.Q., Zhong T. (2024). Improved YOLOv5s small object detection algorithm in UAV view. J. Comput. Eng. Appl..

[30] Zhu X., Lyu S., Wang X., Zhao Q. TPH-YOLOv5: Improved YOLOv5 based on transformer prediction head for object detection on drone-captured scenarios. Proceedings of the IEEE/CVF International Conference on Computer Vision.

[31] Gao T., Li Z., Wen Y., Chen T., Niu Q., Liu Z. (2023). Attention-free global multiscale fusion network for remote sensing object detection. IEEE Trans. Geosci. Remote Sens..

